# Supplementary material for: Medication, age and abstinence days associated with low semen quality: A cross-sectional study in more than 7000 men visiting the centre for reproductive medicine
Source: PLoS One. 2025 Jul 18;20(7):e0326795. doi: 10.1371/journal.pone.0326795 (PMC12273907; doi:10.1371/journal.pone.0326795)

PLOS ONE

Medication, age and abstinence days associated with low semen quality: a cross-sectional study in more than 7000 men visiting the centre for reproductive medicine

--Manuscript Draft--

Manuscript Number:	PONE-D-25-03818
Article Type:	Research Article
Full Title:	Medication, age and abstinence days associated with low semen quality: a cross-sectional study in more than 7000 men visiting the centre for reproductive medicine
Short Title:	Medication and low semen quality
Corresponding Author:	Peter ter Horst, PhD<br>Isala<br>Zwolle, NETHERLANDS, KINGDOM OF THE
Keywords:	medication<br>low semen quality<br>retrospective cohort study
Abstract:	Medication can affect semen quality by decreasing sperm volume, sperm concentration, or decreased sperm motility.<br><br>We performed a retrospective explorative cross-sectional study on medication use and semen quality in our fertility clinic in men older than 18 years with a recorded semen analysis. Men were categorized based on medication use, i.e. any type, and no medication use. Exclusion criteria were incomplete semen analysis, azoospermia, a semen analysis after vasectomy, and days of abstinence less than 2 or more than 7 days before semen collection.<br>The primary outcome was the composite endpoint of low semen quality (LSQ) according to the WHO.<br><br>In total 722 men with medication and 6716 men without medication were included in the study. At the ATC-7 level, univariate (borderline) statistically significant associations using cut-off p<0.100 were found for metformin (A10BA02), metoprolol (C07AB02) and lisinopril (C09AA03), which remained significant after adjusted for age and abstinence days.<br><br>Our study is limited by the fact that information regarding exposure to information, as information was self-reported by patients. Also, the outcome, semen quality, was hindered by patients own collection. Even if a medicament had logically been used for some time before semen sampling (e.g. medication for chronic disease), there was no way to determine whether that medication had been started before or after the occurrence of LSQ. Finally, medication and disease cannot be separated, hence it could not be determined whether medication or the disease itself was associated with LSQ. Therefore, this study should be interpreted as a hypothesis-generating study.
Order of Authors:	Peter Ter Horst, PhD
	Mireille Edens, PhD
	Daphne Den Besten - Bertholee
	Leonie Buurman - Mulder, Msc
	Max Curfs, PhD
Additional Information:	
Question	Response
**Financial Disclosure**<br><br>Enter a financial disclosure statement that describes the sources of funding for the	The author(s) received no specific funding for this work.

Powered by Editorial Manager® and ProduXion Manager® from Aries Systems Corporation

work included in this submission. Review the submission guidelines for detailed requirements. View published research articles from *PLOS ONE* for specific examples.<br><br>This statement is required for submission and **will appear in the published article** if the submission is accepted. Please make sure it is accurate.<br><br>**Funded studies**<br>Enter a statement with the following details:<br>• Initials of the authors who received each award<br>• Grant numbers awarded to each author<br>• The full name of each funder<br>• URL of each funder website<br>• Did the sponsors or funders play any role in the study design, data collection and analysis, decision to publish, or preparation of the manuscript?<br><br>Did you receive funding for this work?	
**Competing Interests**<br><br>Use the instructions below to enter a competing interest statement for this submission. On behalf of all authors, disclose any competing interests that could be perceived to bias this work—acknowledging all financial support and any other relevant financial or non-financial competing interests.<br><br>This statement is required for submission and **will appear in the published article** if the submission is accepted. Please make sure it is accurate and that any funding sources listed in your Funding Information later in the submission form are also declared in your Financial Disclosure statement.<br><br>View published research articles from *PLOS ONE* for specific examples.	The authors have declared that no competing interests exist.

**NO authors have competing interests**<br><br>Enter: *The authors have declared that no competing interests exist.*<br><br>**Authors with competing interests**<br><br>Enter competing interest details beginning with this statement:<br><br>*I have read the journal's policy and the authors of this manuscript have the following competing interests: [insert competing interests here]*<br><br>* typeset	
**Ethics Statement**<br><br>Enter an ethics statement for this submission. This statement is required if the study involved:<br><br>• Human participants<br>• Human specimens or tissue<br>• Vertebrate animals or cephalopods<br>• Vertebrate embryos or tissues<br>• Field research<br><br>Write "N/A" if the submission does not require an ethics statement.<br><br>General guidance is provided below. Consult the submission guidelines for detailed instructions. **Make sure that all information entered here is included in the Methods section of the manuscript.**	Human Subject Research (involving human participants and/or tissue)<br>This study was reviewed by the Medical Ethical Committee of Isala, an independent ethical committee, and gained approval for conduct. Participants were not asked for consent because of the anonimity of the data, and the large number of subjects which is in accordance to Dutch law.

Powered by Editorial Manager® and ProduXion Manager® from Aries Systems Corporation

**Format for specific study types**<br><br>**Human Subject Research (involving human participants and/or tissue)**<br>• Give the name of the institutional review board or ethics committee that approved the study<br>• Include the approval number and/or a statement indicating approval of this research<br>• Indicate the form of consent obtained (written/oral) or the reason that consent was not obtained (e.g. the data were analyzed anonymously)<br><br>**Animal Research (involving vertebrate animals, embryos or tissues)**<br>• Provide the name of the Institutional Animal Care and Use Committee (IACUC) or other relevant ethics board that reviewed the study protocol, and indicate whether they approved this research or granted a formal waiver of ethical approval<br>• Include an approval number if one was obtained<br>• If the study involved *non-human primates*, add *additional details* about animal welfare and steps taken to ameliorate suffering<br>• If anesthesia, euthanasia, or any kind of animal sacrifice is part of the study, include briefly which substances and/or methods were applied<br><br>**Field Research**<br><br>Include the following details if this study involves the collection of plant, animal, or other materials from a natural setting:<br>• Field permit number<br>• Name of the institution or relevant body that granted permission	
**Data Availability**<br><br>Authors are required to make all data underlying the findings described fully available, without restriction, and from the time of publication. PLOS allows rare exceptions to address legal and ethical concerns. See the PLOS Data Policy and FAQ for detailed information.	No - some restrictions will apply

Powered by Editorial Manager® and ProduXion Manager® from Aries Systems Corporation

A Data Availability Statement describing where the data can be found is required at submission. Your answers to this question constitute the Data Availability Statement and **will be published in the article**, if accepted.<br><br>**Important:** Stating 'data available on request from the author' is not sufficient. If your data are only available upon request, select 'No' for the first question and explain your exceptional situation in the text box.<br><br>Do the authors confirm that all data underlying the findings described in their manuscript are fully available without restriction?	
**Describe where the data may be found in full sentences. If you are copying our sample text, replace any instances of XXX with the appropriate details.**<br><br>• If the data are **held or will be held in a public repository**, include URLs, accession numbers or DOIs. If this information will only be available after acceptance, indicate this by ticking the box below. For example: *All XXX files are available from the XXX database (accession number(s) XXX, XXX.*).<br>• If the data are all contained **within the manuscript and/or Supporting Information files**, enter the following: *All relevant data are within the manuscript and its Supporting Information files.*<br>• If neither of these applies but you are able to provide **details of access elsewhere**, with or without limitations, please do so. For example:<br><br>*Data cannot be shared publicly because of [XXX]. Data are available from the XXX Institutional Data Access / Ethics Committee (contact via XXX) for researchers who meet the criteria for access to confidential data.*<br><br>*The data underlying the results presented in the study are available from (include the name of the third party*	The data underlying the results presented in the study are available from Isala (corrsponding author) upon reasonable request

and contact information or URL).<br>• This text is appropriate if the data are owned by a third party and authors do not have permission to share the data.<br><br>* typeset	
Additional data availability information:	

Manuscript

Click here to access/download;Manuscript;manuscript 2024 V3.docx

Medication, age and abstinence days associated with low semen quality: a cross-sectional study in more than 7000 men visiting the centre for reproductive medicine

P.G.J. ter Horst[a, d] , M.A. Edens[b] , D. den Besten-Bertholee[a], L.W. Mulder[a], M.H.J.M. Curfs[c],
a. Department of Clinical Pharmacy, Isala, Zwolle
b. Department of Innovation and Science, Isala, Zwolle
c. Fertility Center Isala, Zwolle
d. Corresponding author. Dr. P.G.J. ter Horst, PhD, hospital pharmacist, dep of Clinical Pharmacy, Isala, Zwolle, The Netherlands. T= 0031 38 424 7244/2469; E=p.g.j.ter.horst@isala.nl, orcid_id: 0000-0003-1914-5907.

Abstract

Medication can affect semen quality by decreasing sperm volume, sperm concentration, or decreased sperm motility.

We performed a retrospective explorative cross-sectional study on medication use and semen quality in our fertility clinic in men older than 18 years with a recorded semen analysis. Men were categorized based on medication use, i.e. any type, and no medication use. Exclusion criteria were incomplete semen analysis, azoospermia, a semen analysis after vasectomy, and days of abstinence less than 2 or more than 7 days before semen collection.
The primary outcome was the composite endpoint of low semen quality (LSQ) according to the WHO.

In total 722 men with medication and 6716 men without medication were included in the study. At the ATC-7 level, univariate (borderline) statistically significant associations using cut-off $p<0.100$ were found for metformin (A10BA02), metoprolol (C07AB02) and lisinopril (C09AA03), which remained significant after adjusted for age and abstinence days.

Our study is limited by the fact that information regarding exposure to information, as information was self-reported by patients. Also, the outcome, semen quality, was hindered by patients own collection. Even if a medicament had logically been used for some time before semen sampling (e.g. medication for chronic disease), there was no way to determine whether that medication had been started before or after the occurrence of LSQ. Finally, medication and disease cannot be separated, hence it could not be determined whether medication or the disease itself was associated with LSQ. Therefore, this study should be interpreted as a hypothesis-generating study.

Competing Interest

None of the authors have anything to declare or have any competing interest.

Key Points

- Semen quality of ~~over~~ 700 men using medication were analyzed compared with semen quality of 6716 men without using medication
- In this hypothesis-generating study metformin, metoprolol and lisinopril were associated with low semen quality

Introduction

Subfertility is defined as the inability of a person to reproduce by natural means. Subfertility is a health issue that affects approximately 15% of couples, who are at their reproductive age, and desiring to have children.[1] This condition is defined by the inability to achieve pregnancy after 1 year of regular unprotected sexual intercourse.[2] Approximately 50% of the subfertility cases are attributed to males.[1] There is evidence that there is a decline in semen quality worldwide and particularly in Western countries which may be related to increased exposure to endocrine disruptors, immunological disorders, and obstruction of the reproductive tract. In addition, unhealthy lifestyles like excessive alcohol consumption and smoking, obesity, and the rising age of reproducing men are suggested.[3,4] Some of these factors also contribute to a decline in the overall health status in the last decades, which causes an increase in the use of medication. Medication, prescribed and non-prescribed, may be associated with a decline in semen quality in men of reproductive age. In the Netherlands, the overall use of prescribed medication in the male population between the ages of 20 - 60 years is approximately 55%.[5–7] Research shows that decreased general health in men together with the use of medication in general may harm semen quality.[8]

Medication can influence semen quality through various mechanisms. Hormonal influence via the hypothalamic-pituitary-testicular axis or by non-hormonal mechanisms, medication may, directly and indirectly, induce impairment of spermatogenesis and alteration of epididymal maturation. Studies have shown that medication affects the impairment of hypothalamic-pituitary-gonadal functions, increases sperm DNA fragmentation and apoptosis, and reduces semen quality.[9] When medication interferes with the endocrine function of the testes by altering Leydig cells or disrupting the hormone regulation system, the drop in testosterone level can affect spermatozoa production.[10]

The mechanism by which medication affects spermatogenesis is unclear for most medications or is derived from data from animal studies. For example, theophylline showed testicular atrophy in rats [11]; a single dose of paracetamol disrupted spermatogenesis at 10 days in rats [12]; morphine in rats resulted in cytogenetic abnormalities in spermatocytes which resolved after 13 weeks of drug withdrawal [13]; a single dose of aspirin disrupted the seminiferous tubule and when it was used for 35 days the total sperm count and motility were decreased.[12] Spironolactone, an aldosterone antagonist, is well known to cause various side effects such as loss of libido and impotence in males.[14]

Information about the safe use of medication related to spermatogenesis is scarce and clinical trials are lacking. Further, most information about medicines and semen quality is derived from animal studies or sparsely from humans. Therefore, this retrospective explorative cross-sectional study aims to investigate the use of medication and possible associations with low semen quality according to the WHO criteria [15] on sperm parameters compared to semen parameters in men without medication use.

METHODS

Study design

We performed a retrospective explorative cross-sectional study on medication use and semen quality.

Ethical approval

The Medical Ethical and Institutional Review Board of Isala gave a waiver for the study protocol on March 12th, 2020 (number 200311). Further, they waived the need for informed consent, as data were anonymized at time of final analysis.

Setting

This study was conducted at the Fertility department of our clinic, a teaching hospital with a large affiliation area. We studied the semen quality of men from couples who visited the fertility department from 2007 to 2018 because of unfulfilled child wish. We accessed all records from the electronic patient files in March 2020 and were able to view identifiable data to retrieve all medical information on our subjects available in the files. We ended data collection on December the 1st 2020.

Study population

All male partners of couples aged older than 18 years with a recorded semen analysis were eligible for inclusion. The indication for semen analysis was a visit to the fertility center because of the couples' infertility. Men were categorized based on medication use, i.e. any type, and no medication use (figure 1). Exclusion criteria were incomplete semen analysis, azoospermia, a semen analysis after vasectomy, and days of abstinence less than 2 or more than 7 days before semen collection.

Outcome, exposure, potential confounders

Outcome

The primary outcome was the composite endpoint of low semen quality, as defined by the WHO laboratory manual for the examination and processing of human semen, 5th edition [15]. This composite endpoint includes (1) sperm volume less than 1.5 mL, or (2) sperm concentration less than 15 million per mL, or (3) total sperm count less than 39 million, or (4) less than 32% progressively motility. If any of these was positive, "low semen quality" (LSQ) was diagnosed, meaning a lower chance of achieving a spontaneous pregnancy was expected.

Semen analysis

The following semen sample data were extracted: ejaculated volume (mL), sperm concentration (10^6/mL), sperm motility parameters (progressive motile, type A and B), non-progressive motile and immotile, type C plus D), total sperm count (10^6/ejaculate), and total motile sperm count (TMSC, 10^6/ejaculate) calculated as the product of ejaculate volume, sperm concentration, and grade A + B motility [15]. Expert laboratory staff of the fertility center at Isala performed all semen analyses.

Medication use Exposure

Medication use of any type was our exposure of interest. The patient self-reported medication use during the time of semen collection was classified according to the Anatomical Therapeutic Chemical (ATC) classification system. Some medications were not specified and could not be classified according to the ATC classification system. Information on dosage and duration was not available and therefore not included.

Potential confounders

We a-priori defined age as a possible confounder. Other potential confounders may be the time of abstinence of ejaculation (less than two or more than 7 days before semen collection); These men, as well as men using multiple medications were excluded from the study.

Data sources and measurement

Data were extracted from electronic patient files of the fertility department.

Patient and public involvement

Patients and public were not involved in the design and conduct of this study. However, the results from this study may be helpful in deciding whether to use specific medication for men trying to inseminate.

Proxy

Medication use was also used as a proxy for conditions/diseases. E.g. men using metformin were considered to have type 2 diabetes mellitus.

Statistical methods

We calculated LSQ prevalence with 95% Wilson confidence intervals, for: 1) all men included in the study, 2) those men on any type of medication(s), and 3) those men using specific medication at ATC code levels 3, 5, and 7. Subsequently, we studied the univariate association of medication use with LSQ using the chi-square test, prevalence difference, prevalence ratio via Poisson regression with robust standard error [16,17], and prevalence odds ratio via logistic regression.[17,18]

Furthermore, we studied the discriminative value of variables for LSQ using receiver operating characteristic (ROC) analysis.

Confounding was considered by studying single medicament users and multivariable analyses. Medications borderline statistically significant ($p<0.100$) were further analyzed by multivariable analysis through Mantel-Haenzel (pooled), Poisson regression with robust standard error, and logistic regression analysis to enable adjustment.

Data analyses were performed using SPSS 28 and STATA 18.

RESULTS

Subject characteristics

Characteristics of the study population

In total 722 men with medication and 6716 men without medication were included in the study. Baseline characteristics of the study group are presented in **table 1**. Men with self-reported medication use were slightly older than men without medication use (mean 34.7±6.4 years and 33.7±5.9 years, respectively). The duration of sexual abstinence was overall 2.8 days in both groups. 54.4% percent of the men with self-reported medication use, used one substance, versus 45.6%% using more than one medication.

Low semen quality

Prevalence and association

Figure 2a shows the LSQ prevalence for all included men and for men on medication(s). Of all n=7273 included men, n=3839 had LSQ, representing an overall LSQ prevalence of 52.8% (95%CI: 51.6% – 53.9%) which is indicated by the horizontal dotted line. Among the n=709 men on medication (any type and any number), we found a higher LSQ prevalence of 56.1% (95%CI: 52.5% – 59.7%). Medication use (any type and any number) was borderline significantly associated with semen quality with p<0.0599 (**figure 2b**) which remained when adjusted for age and abstinence days (**table 2**). Furthermore, LSQ prevalence varied with the number of medications men were taking without a clear trend (**figure 2b**).

Figure 2c shows LSQ prevalence per age category. In the lowest age categories up to 40 years, the LSQ prevalence was more or less around the overall LSQ prevalence of 52.8%. After 40 years, the proportion of LSQ gradually increased to 88.2% in men aged 55 years and older. In men aged 45 years and older, the LSQ prevalence ratio as compared to men younger than 35 was 1.3 with p<0.0001 (**figure 2d**).

Figures 2e and 2f show an inverse association concerning prior semen sample abstinence days with LSQ.

Discrimination

The area under the ROC curve (AUROC) for number of medication (range: 0 to 8) was smaller than the AUROC for age. AUROCs for number of medications, age, and number of abstinence days were 0.480 (95%CI: 0.437 – 0.523, p=0.368), 0.525 (95%CI: 0.512 – 0.538, p<0.001), and 0.469 (95%CI: 0.455 – 0.482, p<0.001), respectively.

Low semen quality per ATC code

Prevalence and association

LSQ prevalences with 95%CIs per ATC level are visualized in **figures 3, 4 and 5**, and plotted against the overall prevalence of 52.8% indicated by the horizontal dotted line. LSQ prevalences were classified into 4 categories: 1] <52.8% (white), 2] 52.8% to <75% (light grey), 3] 75% to <100% (dark grey) and 4] 100% (black) as shown in **figures 3 - 5, and in table 3**.

In many cases LSQ prevalences were high, but sample sizes were too small to reach (borderline) statistical significance, also indicated by wide 95%CIs. In some cases, the combination of prevalence and sample size resulted in a (borderline) statistically significant association, indicated by p-values in red (positive associations) and by p-values in green (inverse association).

Prevalence when using 1 medicament only

Medicaments showing positive (borderline) statistically significant associations were selected for single medicament analysis. When selecting men that reported to use only one medicament, the findings remained largely intact. Some medicamentsshifted to lower or higher classification (**table 3**).

Association

Those ATC codes that showed a (borderline) statistically significant association, and had at least n=10 users, were selected for further statistical analysis (**table 4**). At the ATC-7 level, univariate (borderline) statistically significant associations using cut-off p<0.100 were found for metformin (A10BA02), metoprolol (C07AB02) and lisinopril (C09AA03).

When adjusted for age and abstinence days, these findings remained statistically significant, at least by Poisson analyses.

DISCUSSION

We found several specific medicaments that were associated with a high LSQ prevalence. The numbers of medicament users were however not always considered sufficient for more advanced statistical analysis. Age and the number of abstinence days before semen sample collection were also associated with LSQ, independent of medication use. The study by Halvaei et al. (2020) showed that aging impairs sperm quality, which can affect the fertility status and the outcome of assisted reproductive technologies (Halvaei et al., 2020).
In men visiting our fertility center and using one or more medications, semen quality may be impaired when compared to men without using medication. Especially, the use of metoprolol (beta-blocker), metformin (glucose-lowering drug) and lisinopril (ACE-inhibitor) seems to be associated with low semen quality, independent of age, however, with borderline significance at the p<0.1 level.

We found a positive association between metoprolol and low semen quality, even when adjusted for age. The study by Guo et al showed that the intake of beta-blockers resulted in a lower sperm concentration, motility, and total count [20]. One of the proposed mechanisms by which beta-blockers may induce sexual dysfunction and low semen quality is by inhibiting the sympathetic nervous system, which is involved in the integration of erection, emission, and ejaculation, in the regulation of luteinizing-hormone secretion and the stimulation of release of testosterone [21].
Arterial hypertension and hypercholesterolemia are considered risk factors for hormonal testicular function and spermatogenesis [22]. This risk is clinically relevant since hypertension affects up to 20% of the adult population [23]. The effects of agents acting on the renin-angiotensin system are not yet unequivocally demonstrated. A study by Schill et al. showed improved sperm concentration in men using captopril [24]; however, a retrospective study by Eisenberg et al. concluded that men taking beta-blockers or ACE inhibitors appeared to have a higher risk of infertility, which was not shown in men taking calcium channel blockers [25]. Agents acting on the renin-angiotensin system (enalapril, lisinopril, perindopril, ramipril, losartan, valsartan, candesartan, telmisartan) and sex hormones and modulators of the genital system (testosterone, human chorionic gonadotropin, human menopausal gonadotropin, and clomiphene) also seem to be associated with low semen quality, however, when adjusted for age, the association could no longer be demonstrated. A possible explanation is that older men more often use medication for the treatment of blood pressure control [26]. However, in this study, we could not demonstrate that the use of more than one agent was significantly associated with low semen quality. Further studies are mandatory to explore the role of ACE inhibitors and semen quality.

Most information about the possible effects of medication on semen quality has been obtained from animal studies, and up to now, very little work has been performed to summarize the impact of medication on male fertility except for cancer treatments. A review of Semet et al. looked at the negative impact of pharmacological treatments on male fertility. Medication can impact male fertility by modification of the hypothalamic-pituitary-gonadal axis hormones or by non-hormonal mechanisms.[9] Some medication has a reversible effect on sperm parameters, like sulfasalazine. It has been observed that spermatogenesis generally recovers approximately 2-5 months after stopping sulfasalazine therapy. Testosterone supplementation therapy or anabolic steroids cause azoospermia or severe oligozoospermia. After the withdrawal of exogenous testosterone, spermatogenesis is spontaneously restored after approximately 3 months. Methotrexate, a cytotoxic agent that is also indicated for numerous autoimmune conditions, is gonadotoxic, mutagenic, and probably teratogenic, but its effects are reversed after the discontinuation of treatment [10].

In this study we used the prevalence ratio's and odds ratio's, as measures of association based on prevalence of low semen quality.[27] The prevalence ratios may prevent the overestimation of odds ratio's of the outcome as was demonstrated by Tamhane et al. (2016) and may be more accurate.[28]

Our study is limited, inherent to the study design, by the fact that information regarding exposure to information, as information was self-reported by patients. Also, the outcome, semen quality, was hindered by patients own collection. Therefore, caution is needed in concluding. Even if a medicament had logically been used for some time before semen sampling (e.g. medication for chronic disease), there was no way to determine whether that medication had been started before or after the occurrence of LSQ. Therefore, this study should be interpreted as a hypothesis-generating study. Finally, medication and disease cannot be separated, hence it could not be determined whether medication or the disease itself was associated with LSQ.

Conclusion

We found that the use of metoprolol (beta-blocker), metformin (glucose-lowering drug) and lisinopril (ACE-inhibitor) seem to be associated with low semen quality, independent of age.

Declarations

Funding
Not applicable

Conflict of interest
None of the authors has any kind of conflict of interest

Availability of data and materials
Data are available upon reasonable request to the corresponding author

Ethics approval
The Medical Ethical Committee of Isala gave a waiver for the conduct of this study

Author contributions
All authors made substantial contributions to the conception and design of the work; revised it critically for important intellectual content; approved the version to be published; agree to be accountable for all aspects of the work in ensuring that questions related to the accuracy or integrity of any part of the work are appropriately investigated and resolved. PTH and ME were responsible for analysis, or interpretation of data.

References

1. Henkel R, Offor U, Fisher D. The role of infections and leukocytes in male infertility. Andrologia. Blackwell Publishing Ltd; 2021.
2. Thurston L, Abbara A, Dhillo WS. Investigation and management of subfertility. J Clin Pathol. 2019;72:579–87.
3. Hammiche F, Laven JSE, Boxmeer JC, Dohle GR, Steegers EAP, Steegers-Theunissen RPM. Sperm quality decline among men below 60 years of age undergoing IVF or ICSI treatment. J Androl. 2011;32:70–6.
4. Wong WY, Thomas CMG, Merkus HMWM, Zielhuis GA, Doesburg WH, Steegers-Theunissen RPM. Cigarette smoking and the risk of male factor subfertility: Minor association between cotinine in seminal plasma and semen morphology. Fertil Steril. 2000;74:930–5.
5. Otte-Huijgen N, Goijen HJ ~~(Hedwig J).~~, Twigt J, Mulders A, Lindemans J, Dohle G, et al. Effect of Medications for Gastric Acid-Related Symptoms on Total Motile Sperm Count and Concentration: A Case–Control Study in Men of Subfertile Couples from the Netherlands. Drug Saf. 2017;40:241–8.
6. Electronic Databank of Statistics Netherlands: Medication use in men of reproductive age (www.cbs.nl). 2021.
7. Pompe S V, Strobach D, Stief CG, Becker AJ, Trottmann M. Drug use among men with unfulfilled wish to father children: a retrospective analysis and discussion of specific drug classes. Pharmacoepidemiol Drug Saf. 2016;25:668–77.
8. Ventimiglia E, Capogrosso P, Boeri L, Serino A, Colicchia M, Ippolito S, et al. Infertility as a proxy of general male health: Results of a cross-sectional survey. Fertil Steril. 2015;104:48–55.
9. Ajayi AF, Akhigbe RE. The physiology of male reproduction: Impact of drugs and their abuse on male fertility. Andrologia. Blackwell Publishing Ltd; 2020.
10. Semet M, Paci M, Saïas-Magnan J, Metzler-Guillemain C, Boissier R, Lejeune H, et al. The impact of drugs on male fertility: a review. Andrology. Blackwell Publishing Ltd; 2017. p. 640–63.
11. Friedman L, Weinberger M, Farber T, Moreland F, Peters E, Gilmore C, et al. Testicular atrophy and impaired spermatogenesis in rats fed high levels of the methylxanthines caffeine, theobromine, or theophylline. J Environ Pathol Toxicol. 1979;2:687–706.
12. Ekaluo U, Ikpeme E, Udokpoh A. Sperm head abnormality and mutagenic effects of aspirin, paracetamol and caffeine containing analgesics in rats. The Internet Journal of Toxicology. 2008;7.
13. James RW, Heywood R, Crook D. EFFECTS OF MORPHINE SULPHATE ON PITUITARY-TESTICULAR MORPHOLOGY OF RATS. Toxicol Lett. 1980.
14. Miyatake A, Noma K, Nakao K, Morimoto A N D Y, Yamamura Y. INCREASED SERUM OESTRONE A N D OESTRADIOL FOLLOWING SPIRONOLACTONE ADMINISTRATION IN HYPERTENSIVE MEN. 1978.
15. WHO World Health Organization. WHO Laboratory Manual for the Examination and Processing of Human Semen. 5th ed. Geneva, Switzerland: World Health Organization Department of Reproductive Health and Research. 2010.
16. S Coutinho I LM, Rossi Menezes P. Methods for estimating prevalence ratios in cross-sectional studies. Rev Saúde Pública. 2008.
17. Behrens T, Taeger D, Wellmann J, Keil U. Different MethodstoCalculate Effect Estimates in Cross-sectional Studies AComparison between Prevalence Odds Ratio and Prevalence Ratio.
18. Reichenheim ME, Coutinho SF. Measures and models for causal inference in cross-sectional studies: arguments for the appropriateness of the prevalence odds ratio and related logistic regression [Internet]. 2010. Available from: http://www.biomedcentral.com/1471-2288/10/66
19. Halvaei I, Litzky J, Esfandiari N. Advanced paternal age: effects on sperm parameters, assisted reproduction outcomes and offspring health. Reproductive Biology and Endocrinology. BioMed Central Ltd; 2020.
20. Guo D, Li S, Behr B, Eisenberg ML. Hypertension and Male Fertility. World J Mens Health. 2017;35:59.
21. Fogari R, Preti P, Derosa G, Marasi G, Zoppi A, Rinaldi A, et al. Effect of antihypertensive treatment with valsartan or atenolol on sexual activity and plasma testosterone in hypertensive men. Eur J Clin Pharmacol. 2002;58:177–80.
22. Lorenzini F. Beneficial effects of Renin-Angiotensin system blockers on testicular steroidogenesis. Journal of Urology. 2014;12:1883.
23. Alves-Pereira J, Frantz E, da Fonte Ramos C. Beneficial effects of Renin-Angiotensin system blockers on testicular steroidogenesis. Journal of Urology. 2014;192.
24. Schill W, Parsch E, Miska W. Inhibition of angiotensin-converting enzyme—a new concept of medical treatment of male infertility? Fertil Steril. 1994;61:1123–8.

25. Eisenberg M, Li S. Special research presentation: The association between antihypertensives and male infertility using insurance claims data. In: Drobnis E, Nangia A, editors. Impacts of Medications on Male Fertility. Springer; 2017.
26. König M, Gollasch M, Rosada A, Demuth I, Spira D, Steinhagen-Thiessen E. Antihypertensive Treatment Patterns and Blood Pressure Control in Older Adults: Results from the Berlin Aging Study II. Drugs Aging. 2018;35:993–1003.
27. Ahrens W, Pigeot I. Handbook of Epidemiology. Bremen, germany; 2014.
28. Tamhane AR, Westfall AO, Burkholder GA, Cutter GR. Prevalence odds ratio versus prevalence ratio: choice comes with consequences. Stat Med. 2016;35:5730–5.

Figure1

Click here to access/download;Figure;figure 1 flow chart in and excluded patients.pptx

Figure 1: Flowchart of in- and excluded patients

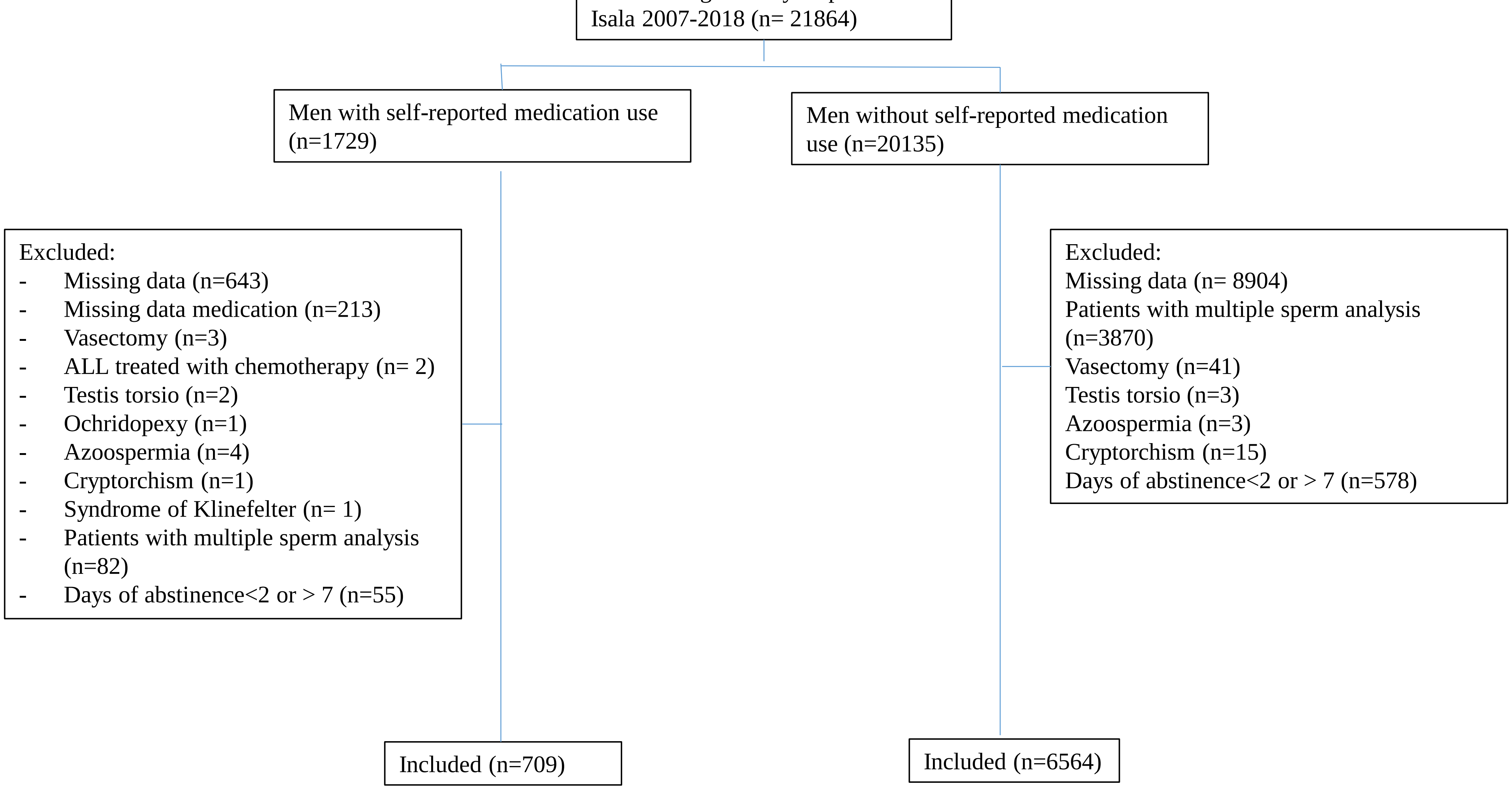

Figure2

Click here to access/download;Figure;Figure 2_Overview _ 20241124 _ w15h8,dpi1000.pdf

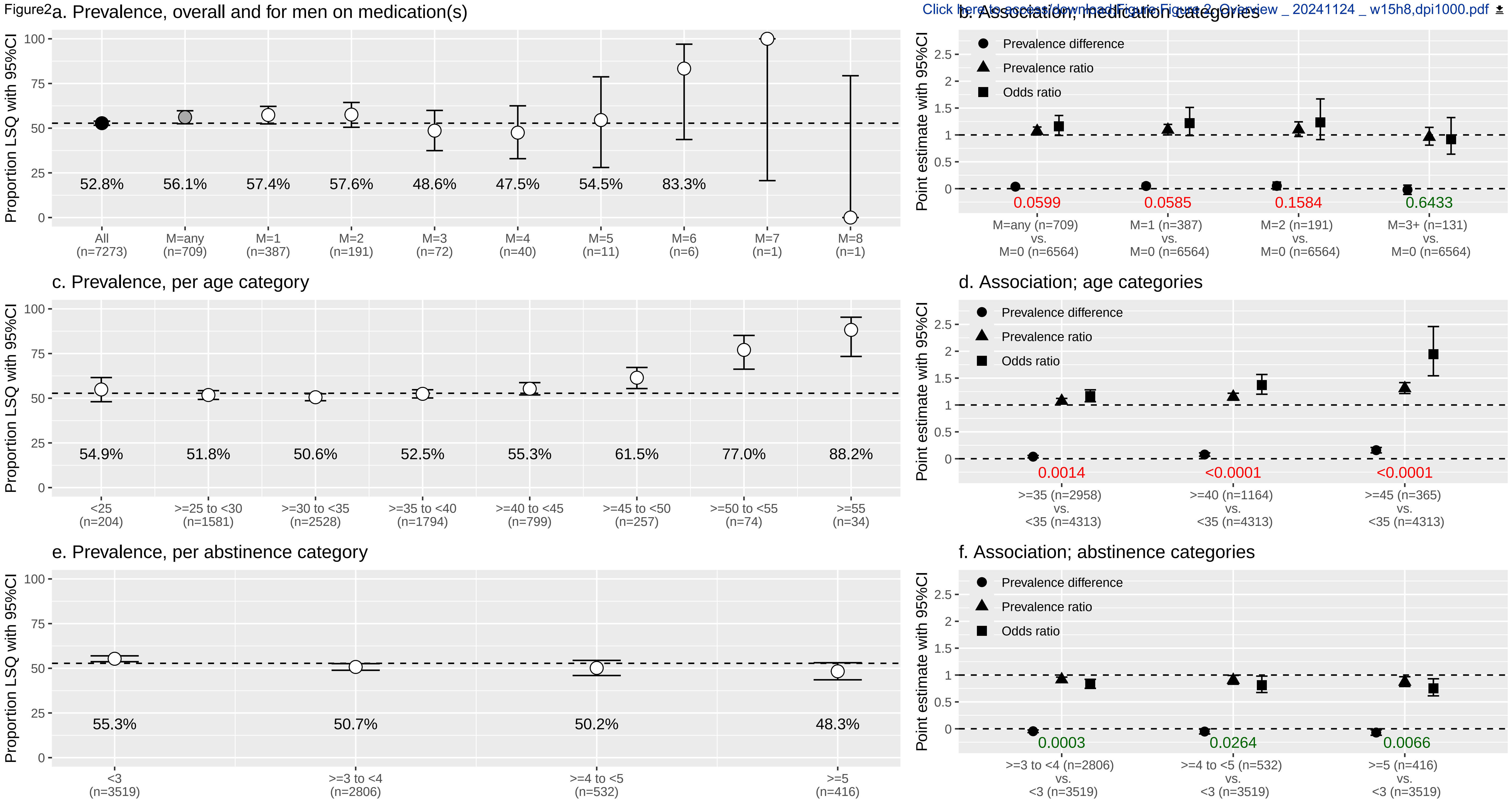

Figure3

Click here to access/download;Figure;Figure 3. ATC3 LSQ_20241124_dpi1000.pdf

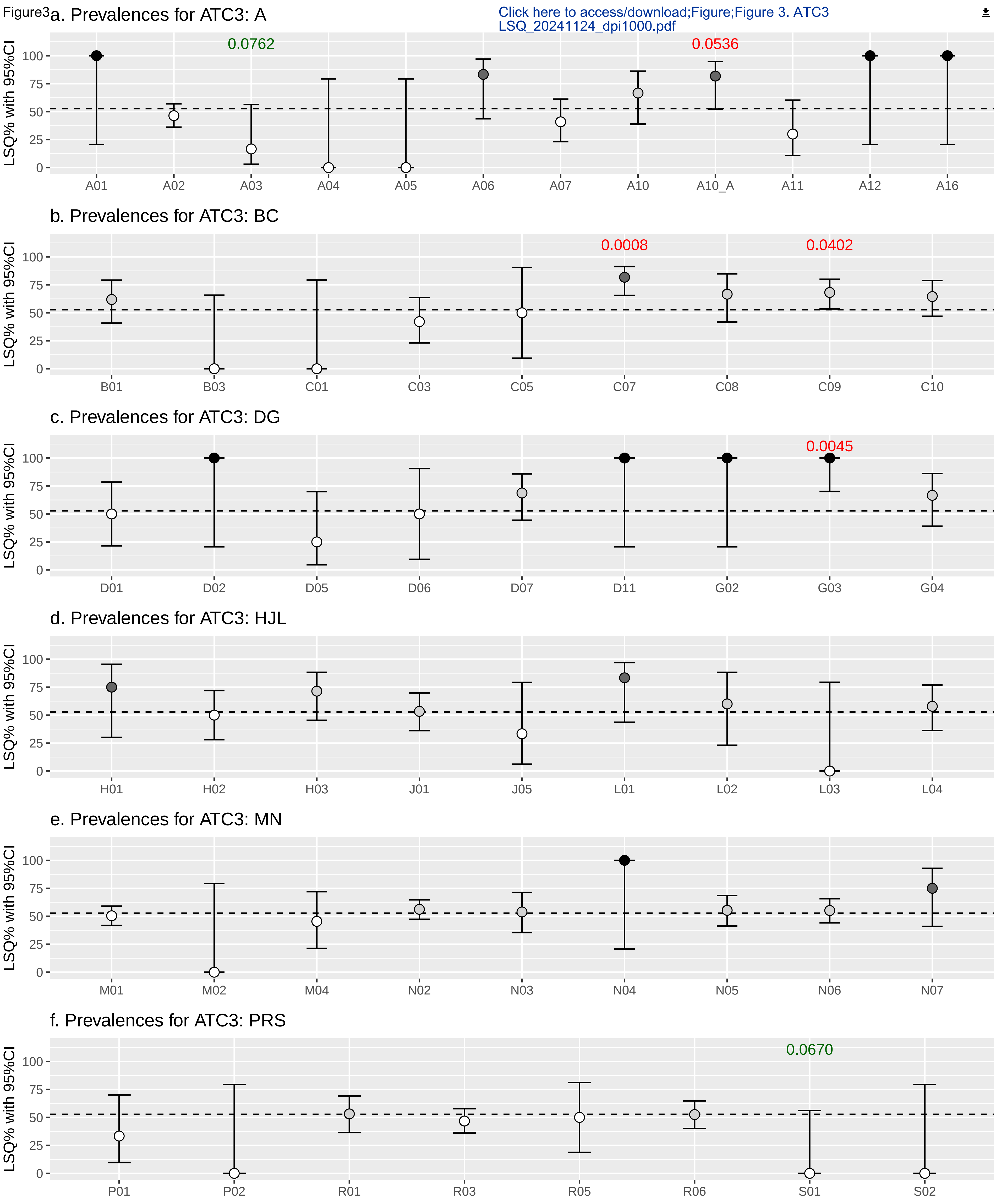

Figure4

Click here to access/download;Figure;Figure 4. ATC5 LSQ_20241124_dpi1000.pdf

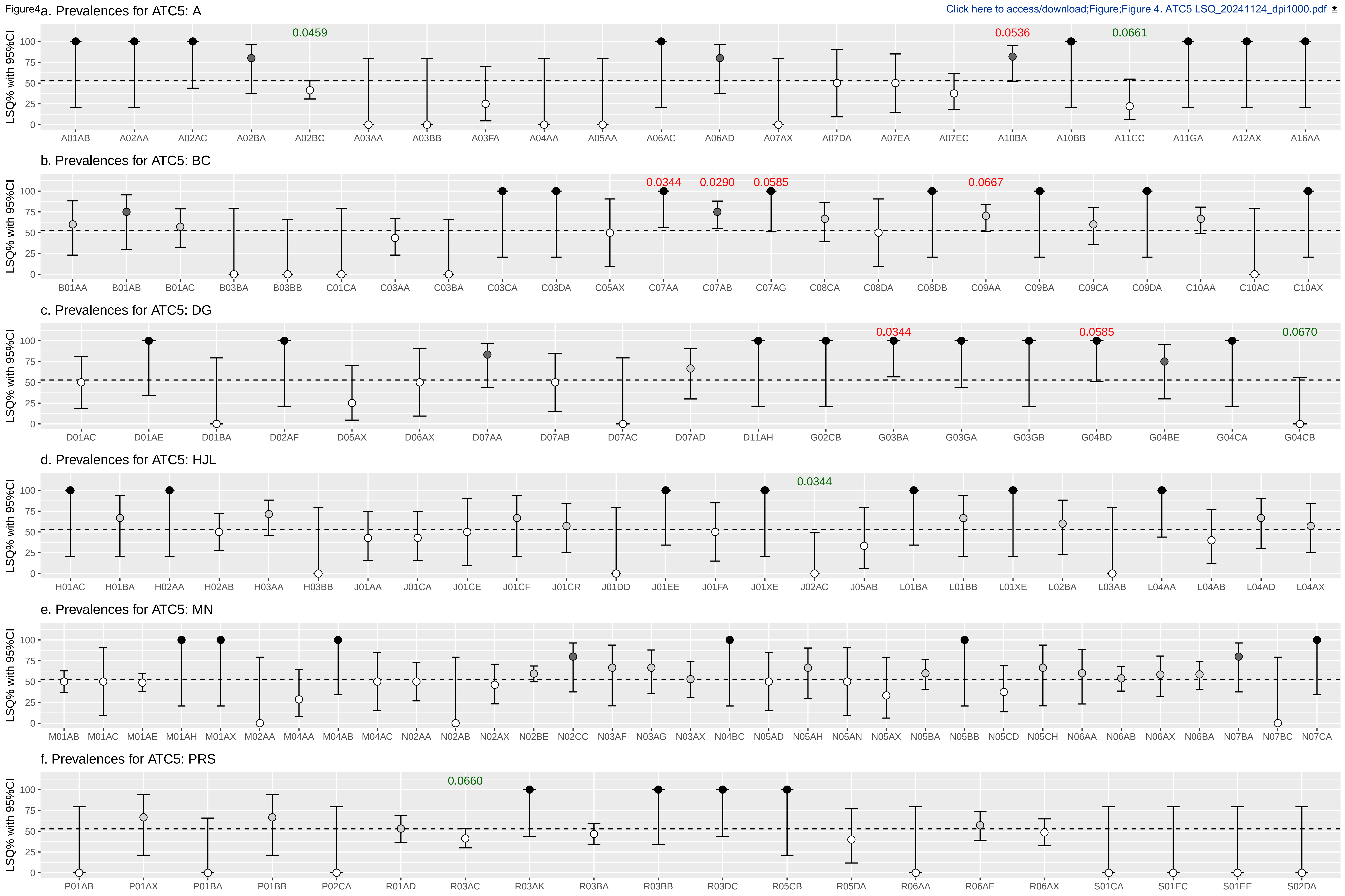

Click here to access/download;Figure;Figure 5. ATC7 LSQ_20230424_dpi1000.pdf

Figure5

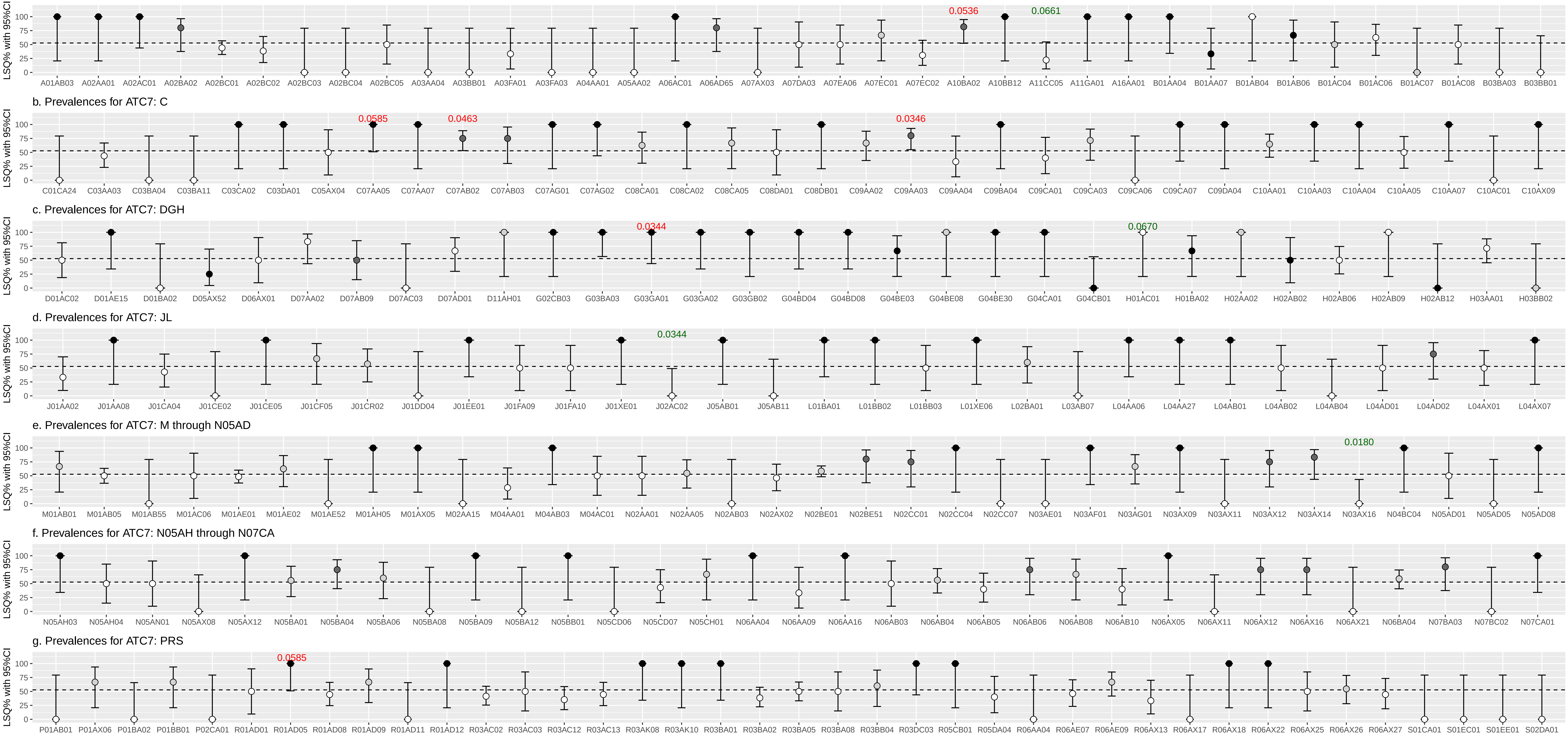

Supplement: Attachment — Submitted filename: PONE-D-25-03818_reviewer_respons.pdf [file pone.0326795.s004.pdf]
